# Developing Tadpole *Xenopus laevis* as a Comparative Animal Model to Study *Mycobacterium abscessus* Pathogenicity

**DOI:** 10.3390/ijms22020806

**Published:** 2021-01-15

**Authors:** Arianna Lopez, Carolyn Shoen, Michael Cynamon, Dionysia Dimitrakopoulou, Matthieu Paiola, Martin S. Pavelka, Jacques Robert

**Affiliations:** 1 Department of Immunology and Microbiology, Medical Center, University of Rochester, Rochester, NY 14642, USA; alopez17@u.rochester.edu (A.L.); Dionysia_Dimitrakopoulou@URMC.Rochester.edu (D.D.); Matthieu_Paiola@URMC.Rochester.edu (M.P.); Martin_Pavelka@urmc.rochester.edu (M.S.P.J.); 2 Central New York Research Corporation, Syracuse, NY 13210, USA; Carolyn.Shoen@va.gov (C.S.); Michael.Cynamon@va.gov (M.C.); 3 Veterans Affairs Medical Center, Syracuse, NY 13210, USA

**Keywords:** morphotype, dissemination, microbial persistence, Xenopus, mycobacteria, non-mammalian model, aquatic vertebrates, larval stage

## Abstract

*Mycobacterium abscessus (Mab)* is an emerging, nontuberculosis mycobacterium (NTM) that infects humans. *Mab* has two morphotypes, smooth (S) and rough (R), related to the production of glycopeptidolipid (GPL), that differ in pathogenesis. To further understand the pathogenicity of these morphotypes in vivo, the amphibian *Xenopus laevis* was used as an alternative animal model. *Mab* infections have been previously modeled in zebrafish embryos and mice, but *Mab* are cleared early from immunocompetent mice, preventing the study of chronic infection, and the zebrafish model cannot be used to model a pulmonary infection and T cell involvement. Here, we show that *X. laevis* tadpoles, which have lungs and T cells, can be used as a complementary model for persistent *Mab* infection and pathogenesis. Intraperitoneal (IP) inoculation of S and R *Mab* morphotypes disseminated to tadpole tissues including liver and lungs, persisting for up to 40 days without significant mortality. Furthermore, the R morphotype was more persistent, maintaining a higher bacterial load at 40 days postinoculation. In contrast, the intracardiac (IC) inoculation with S *Mab* induced significantly greater mortality than inoculation with the R *Mab* form. These data suggest that *X. laevis* tadpoles can serve as a useful comparative experimental organism to investigate pathogenesis and host resistance to *M. abscessus*.

## 1. Introduction

*Mycobacterium abscessus (Mab)* is a nontuberculosis mycobacterium (NTM) commonly found in water and soil [1,2,3]. This emerging pathogen can infect multiple human tissues including skin, lungs, and other vital organs [2,4]. *Mab* has become a particular threat to humans with chronic lung diseases such as bronchiectasis and cystic fibrosis as well as immunocompromised patients [5,6,7]. In fact, *Mab* is the second most prevalent cause of NTM pulmonary infections in the United States, causing 2.6–13.0% of all mycobacterial pulmonary infections [8]. However, *Mab* can also cause cutaneous infections in healthy individuals [2,5]. Treatment of infections caused by *Mab* is challenging due to its multiantibiotic resistance and the ability of this pathogen to evade the host’s immune system, aiding in its persistence [5]. In addition, determining the mechanisms by which *Mab* causes disease can be used to better understand the pathogenicity of *Mycobacterium tuberculosis (Mtb).* Both pathogens share multiple physiological features [9]. As such, *Mab* can be used to better understand the virulence mechanisms that *Mtb* presents since *Mab* has a lower Biohazard Safety Level and grows faster than *Mtb* in simple media [10].

Beyond the plasma membrane, all mycobacteria have a complex mycolyl–arabinogalactan–peptidoglycan cell wall with a second lipid bilayer, the mycomembrane, distal to the wall [11]. *Mab* has two morphotypes, smooth (S) and rough (R), which are due to the presence of glycopeptidolipid (GPL), a structure found on the outer leaflet of the mycomembrane [12]. The R morphotype has low, or lacks, GPL production, resulting in the formation of dry colonies in vitro on agar plates, while the S morphotype has high production of GPL, resulting in the formation of circular, moist colonies [13]. It has been shown that the R morphotype derives from a S precursor [14]. Multiple genes are responsible for the assembly and transport of GPL across the plasma membrane and cell wall; therefore, a deletion mutation in any one of these genes could hinder or completely arrest the production of GPL. A large fraction of R variant clinical isolates is due to single nucleotide deletions or insertions within two genes: *mmpl4* or *mps1* [12]. These two genes, located within the GPL locus, play essential roles for GPL synthesis but are not essential for the organism’s survival [15].

Epidemiological surveys have revealed that R *Mab* is more prominent in patients with severe pulmonary infections [1,3,12]. The S morphotype, which is commonly isolated from nature, may be acquired by susceptible individuals through hospital contamination, drinking water, or soil [12]. However, once an infection with S *Mab* is acquired, a S to R switch may occur. A spontaneous S to R switch has a frequency of approximately 1 in 10^5^ to 1 in 10^6^ [13]. The preferential selection of the R morphotype has been observed in many clinical isolates with and without cystic fibrosis [16].

GPL impacts many aspects of pathogenicity by changing sliding mobility, bacteria interaction, and biofilm formation [12,17]. High GPL production is associated with increased sliding mobility and biofilm formation as well as tolerance to acidic pH [12,17]. In contrast, low GPL production promotes aggregation and cording [12,13,17]. Cords are irregular filaments that form ridges and extend outward [13]. It is noteworthy that cords produced by R *Mab* are morphologically similar to those produced by *Mtb* [13]. S *Mab* lack the ability to form cords because GPL blocks the interaction of trehalose dimycolate (TDM) molecules, which are involved in the cord formation mechanism [13,17]. The rough morphotype’s cording ability and aggregation properties may increase its pathogenesis in the host environment, leading to a more persistent and invasive infection.

The pathogenicity of R and S morphotypes remains poorly understood. In fact, studies on the host–pathogen interaction remain fragmented and inconsistent. The differential expression of GPL appears to affect interactions with host cells, including phagocytosis and immune reaction. Although the R morphotype is considered hypervirulent, inducing tumor necrosis factor (TNF) production and phagocytosis through toll-like receptor 2 (TLR2) signaling, the immune response of S *Mab* is not clearly characterized [18]. If GPL is highly immunogenic, inflammation and innate immune cell recruitment caused by GPL may promote the dissemination of the pathogen [12]. During the early stages of infection, GPL continues to induce the production of cytokines and chemokines, promoting the recruitment of macrophages and additional neutrophils [12]. Neutrophils are only able to kill approximately 25% of the *Mab* by phagocytosis and neutrophil extracellular traps (NETs) [19]. This may select for the survival of R, GPL-deficient, variants and mark the transition from S to R morphotypes during *Mab* infection, which would drive colonization toward persistence and invasion of host tissues (19). Furthermore, it has been reported that the presence of GPL could mask ligands that bind to macrophage surface receptors, preventing phagocytosis [20]. In contrast, toll-like receptor 2 (TLR-2) has been shown to bind phosphatidyl-myo-inositol mannoside (PIMs), a ligand on the outer surface of R *Mab,* to signal phagocytosis [21]. *Mab* is an intracellular pathogen; therefore, macrophage engulfment may be beneficial for replication. In addition, R *Mab* may be more equipped to survive phagocytosis due to its ability to aggregate and form cords. Aggregation upon phagocytosis leads to the formation of phagocytic cups resulting in phagocyte cell death [22].

For in vivo studies, *Mab* infections have been modeled using zebrafish embryos and mice [12]. Studies in mice have been hampered by a lack of persistent infection with *Mab*, as immunocompetent mice clear the pathogen within the first couple of weeks postinfection [12]. Moreover, TNFR^−/−^, Cybb^−/−^, and Nos2^−/−^ mice were all able to completely clear *Mab* in the lungs, spleen, and liver 40 days after intravenous injection of 1 × 10^6^ CFUs [23]. Even though these mice lacked an intact innate immune system, the infection triggered an innate response one day postinfection, resulting in early clearance [23]. *Mab* clearance was less efficient in MyD88^−/−^ and GKO^−/−^ mice, with 3.5 log_10_ CFUs *Mab* at 40 days postinfection [23].

The attractiveness of the zebrafish model includes a convenient visualization of host–pathogen interactions because their embryos are transparent [12]. However, there are some limitations of this model, including the lack of lungs, which are necessary to model pulmonary infections, and the lack of functional adaptive immunity during the early stages of development [12]. Infection of zebrafish embryos with the R morphotype resulted in 65% mortality by 13 days postinfection, whereas less than 10% of the S-infected embryos died by 13 days postinfection [22]. There was no difference in the phagocytosis of the S and R morphotypes [9]. Engulfment did initially limit the pathogen’s growth, but ultimately enhanced the spread of the microbe to tissues beyond the site of infection. The innate mechanisms of macrophages only protected the host from infection with the S morphotype [9]. The R morphotype was more pathogenic because, at the site of infection, the bacteria were immediately engulfed by macrophages. Additional macrophages were recruited to the infection site and caused granuloma formation [9]. Phagocytized bacteria then migrated via the circulatory system to other tissues. Intracellular growth and cord formation triggered macrophage apoptosis, which released the bacteria [9]. Further cord formation prevented repeated phagocytosis, resulting in replication of the bacteria and tissue damage [9]. Altogether, these studies in murine and zebrafish experimental organisms show numerous inconsistencies indicating that the host–pathogen interactions in *Mab* are complex and would benefit from the development of complementary animal models.

*X. laevis* tadpoles have lungs that become functional for breathing between four and five days postfertilization, which permits the study of pulmonary infections [24]. Furthermore, like zebrafish embryos, *X. laevis* tadpoles are transparent, allowing visualization of pathogen dissemination, while their immune system is more similar to that of humans, including the adaptive arm. Previous research has concluded that the mammalian and *Xenopus* adaptive and innate immune systems are evolutionarily conserved based on extensive similarity [25]. We have previously developed a *Mycobacterium marinum* infection model with *X. laevis* and hypothesized that this model could also be used to study *Mab* pathogenesis [26].

The purpose of this study was to evaluate whether the amphibian *X. laevis* tadpole could provide a reliable model relevant to humans for studying *Mab* infection by comparing the survival of the host and the survival and proliferation of S and R *Mab.* Since R and S *Mab* present different structural surface ligands, we postulate that different immune responses to these morphotypes should occur resulting in distinct survival rates in infected tadpoles. Our results provide new in vivo evidence of differences in the pathogenesis of S and R *Mab* variants and establish *X. laevis* tadpoles as a useful new comparative experimental organism to examine host interaction with *Mab*.

## 2. Results

### 2.1. Clearance of Mab in Mice

**Figure 1:**
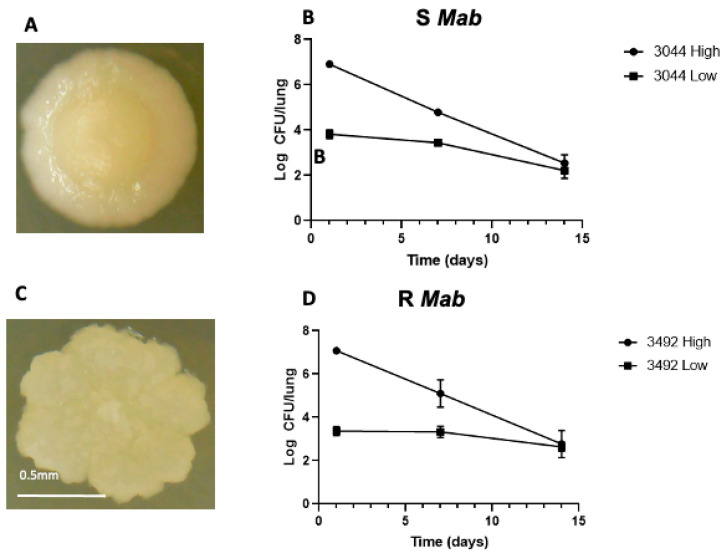
Colony morphology and bacterial abundance in C57BL/6 mice 1, 7, and 14 dpi. (**A**) Picture of a smooth (S) *Mab* colony cultured 5 days at 37 °C on agar plates showing a circular shape and a moist appearance; (**C**) Rough (R) *Mab* colony with rugged edges and dry appearance. Mice were infected with high (10^7^ CFUs) and low (10^4^ CFUs) of (**B**) S (3044) and (**D**) R (3492) *Mab* by intranasal infection. The right lung was collected from four mice per timepoint, and lysates were plated on agarose media. Average bacterial load decreased from 1 to 14 days postinfection (dpi) for each morphotype and dose.

We confirmed that our S and R *Mab* strains are cleared in the mouse model as expected. After intranasal challenge of C57BL/6 mice with high (10^7^ CFUs) and low (10^4^ CFUs) doses, each morphotype was cleared, with a faster decrease seen in the high dose experiments (Figure 1). Unlike zebrafish, where increasing the dose of the rough morphotype led to increased mortality [22], high (10^7^ CFUs) and low (10^4^ CFUs) doses of S and R *Mab* resulted in a reduction in concentration in the lungs to less than 10^3^ CFUs 14 days postinfection.

### 2.2. Dissemination and Persistence of S and R Mab in Intraperitoneally Infected X. laevis Tadpoles

**Figure 2:**
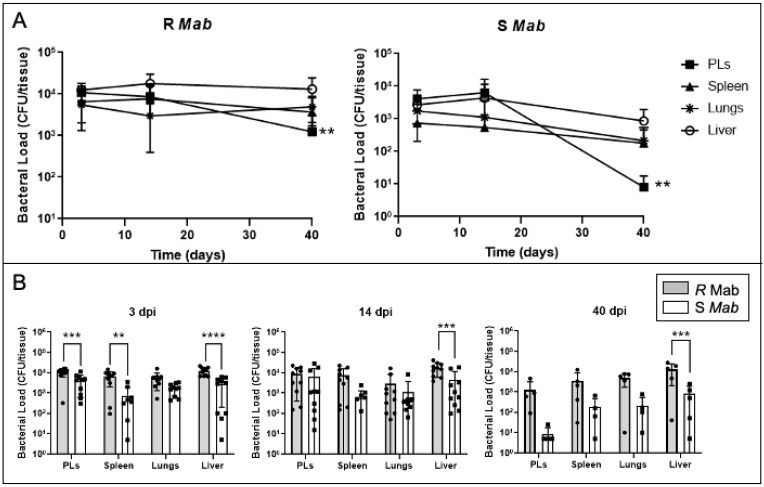
Bacterial recovery from tadpoles 3, 14, and 40 days after intraperitoneal (IP) inoculation of either R or S *Mab*. (**A**) Timeline and (**B**) two-by-two comparison. Tadpoles were IP-injected with 5 × 10^5^ CFU in a 10 μL volume. Spleen, liver, peritoneal leukocytes (PLs), and lungs were collected, suspended in 500 µL of aPBS, lysed by bead beating, and plated on agar media. The bacterial abundance was determined by the number of CFUs/tissue. **p < 0.005, ***p < 0.0005, and ****p < 0.0001 (two-way ANOVA followed by post hoc analysis using Tukey’s multiple comparisons test).

To determine whether S and R *Mab* strains can infect *X. laevis* tadpoles, we followed the same intraperitoneal (IP) inoculation procedure that we previously used with *M. marinum* [26]. Following the injection of 5 × 10^5^ CFUs of *Mab*, we used bacterial plate counts to determine the abundance of S and R *Mab* in lysates of different *X. laevis* tissues 3 days postinfection (dpi), 14 dpi, and 40 dpi (Figure 2A). We observed wide dissemination and persistence of both S and R Mab since significant CFU were recovered from all the tissues tested until 40 dpi. On average, 10^3^ and 10^4^ R *Mab* CFU/tissue were recovered at each time point. More detailed comparisons between S and R *Mab* (Figure 2B), indicates that tadpoles infected with S *Mab* presented fewer CFU in lysates across tissues and time points, between 10^1^ and 10^3^ CFU/tissue, with declining bacterial abundance from 3 to 40 dpi. Notably, significantly less S than R *Mab* were recovered from the liver at the three time points, and very low CFU counts were obtained for peritoneal leucocytes (PLs) at 40 dpi. In conclusion, a relatively modest dose of either S and R *Mab* inoculum can infect, disseminate, and persist in tadpoles, while R *Mab* can persist at higher loads than S *Mab* within multiple organs.

### 2.3. Survival Analysis of Tadpole X. laevis Inoculated with S or R Mab

**Figure 3:**
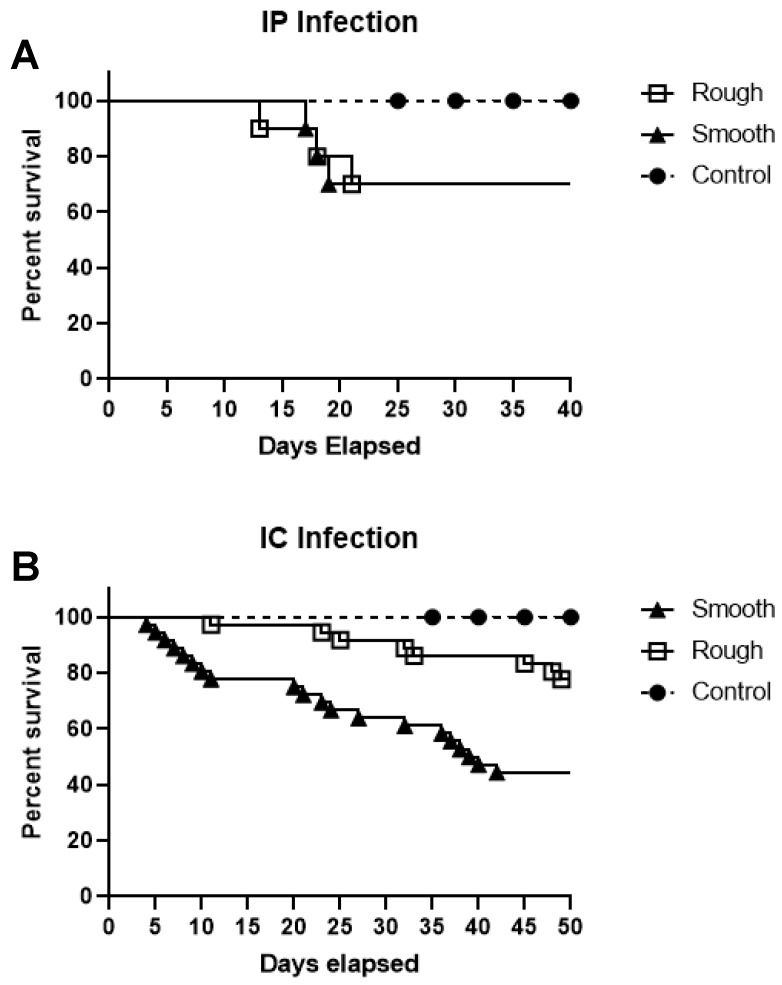
Tadpole survival following IP (**A**) or IC (**B**) inoculation with either R or S *Mab*. (**A**) Twenty tadpoles, ten in each group, were inoculated with 5 × 10^5^ CFU of R or S *Mab* by IP infection and monitored for 40 days. (**B**) Seventy-two tadpoles were inoculated with 5 × 10^5^ CFU of R or S *Mab* by IC infection and monitored for 50 days for survival. There was a significant difference in the survival of S and R IC inoculated tadpoles (*p* < 0.005) based on the log-rank (Mantel–Cox) test.

To examine tadpole host resistance to S and R *Mab,* survival assays were performed using 5 × 10^5^ CFU of *Mab* by IP inoculation. Over the course of 40 days, only modest mortality (30%) was associated with S and R *Mab* infection (Figure 3A), and survivorship was not affected by the *Mab* morphotype (*p* = 0.4983).

The apparent tadpole resistance to S and R *Mab* infection, despite the wide dissemination and persistence of the pathogens, prompted us to determine whether tadpole survival would be affected by another route of inoculation. To be close to an intravenous inoculation resulting in a rapidly spreading infection in the whole organism, we decided to use intracardiac (IC) injection, which is well tolerated in tadpoles. Notably, tadpoles were found to be more susceptible to S *Mab* infection following IC inoculation compared to IP inoculation of 5 × 10^5^ CFUs of bacteria (Figure 3B). Indeed, tadpoles infected with S *Mab* started to die at 4 dpi, whereas all control tadpoles injected with aPBS survived the duration of the 50-day study. The median survival of tadpoles infected with S *Mab* was 39.5 days. In contrast, only 1 tadpole infected with R *Mab* died at 8 dpi, and less than 10 died over the 50-day period, which suggests that tadpoles are more resistant or tolerant to R *Mab*.

### 2.4. Dissemination and Persistence of S and R Mab in IC-Infected X. laevis Tadpoles

**Figure 4:**
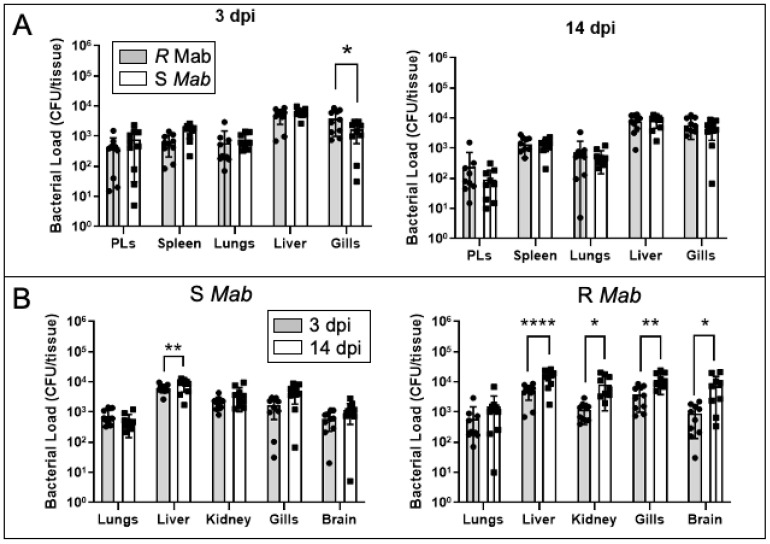
Bacterial recovery from tadpoles 3 and 14 days after intracardiac (IC) inoculation of either R or S *Mab*. (**A**) Comparison of R and S *Mab* CFUs for different tissues at 3 and 14 dpi. (**B**) CPU comparison between 3 and 14 dpi for R and S *Mab.* Tadpoles were IC-injected with 5 × 10^5^ CFU in a 10 μL volume. Spleen, liver, PLs, and lungs were collected, suspended in 500 µL of aPBS, lysed by bead beating, and plated on agar media. The bacterial abundance was determined by the number of CFUs/tissue. * *p* < 0.05, ** *p* < 0.005, and **** *p* < 0.0001 (two-way ANOVA followed by post hoc analysis using Tukey’s multiple comparisons test).

Since the intracardiac route of *Mab* inoculation induced higher mortality in tadpoles infected with the S morphotype, we tested whether this was associated with higher bacterial loads and/or differences in dissemination. We assayed bacterial burden at 3 dpi and 14 dpi and expanded the bacterial recovery to include gills, kidney, and brain, in addition to the liver, spleen, PLs, and lungs (Figure 4A). Overall, CFU recovered at 3 and 14 dpi were comparable between S and R morphotypes, with the exception of the gills that exhibited significantly lower S *Mab* load at 3 dpi. We also noted an increase in bacterial loads for both morphotypes in the liver for the two time points. When comparing CFUs between 3 and 14 dpi, there was a consistent expansion (1 log on average) of R *Mab* in most tissues, whereas S *Mab* CFUs remained stable (Figure 4B).

**Figure 5:**
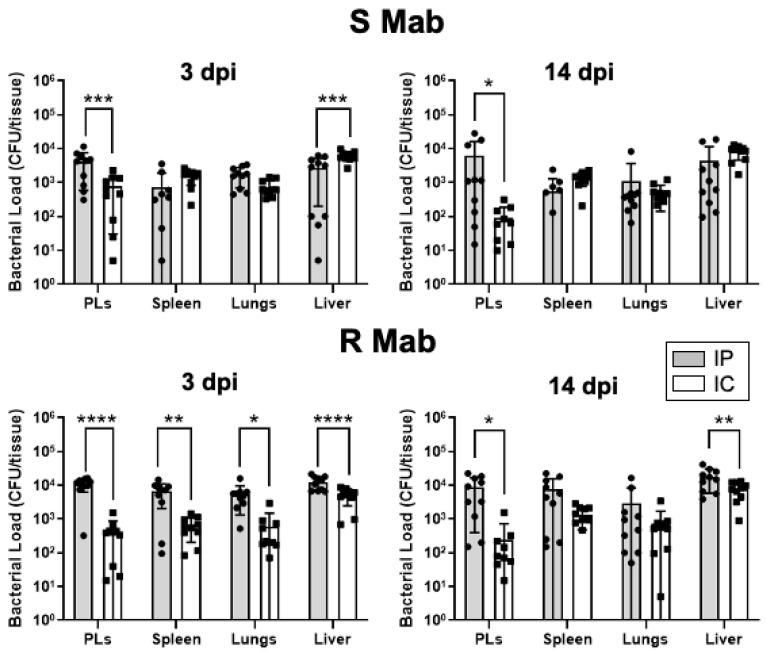
Comparison of bacterial recovery between IP and IC inoculation at 3 and 14 dpi for S and R *Mab*. * *p* < 0.05, ** *p* < 0.005, *** *p* < 0.0005, and **** *p* < 0.0001 (two-way ANOVA followed by post hoc analysis using Tukey’s multiple comparisons test).

Because of the difference in tadpole survival, we also compared the bacterial loads recovered following IP versus IC inoculation (Figure 5). Early establishment and dissemination of R *Mab* at 3 dpi by the IP route were significantly higher than the IC route, and this difference persisted at 14 dpi. In contrast, except for PLs where lower S *Mab* loads were detected at 3 and 14 dpi, comparable dissemination was detected in other organs.

## 3. Discussion

The goal of this study was to evaluate the potential of *X. laevis* tadpole to serve as a useful comparative experimental organism alternative to mice and zebrafish to investigate *Mab* pathogenesis and host–pathogen interactions. Our data show that both S and R *Mab* morphotypes can readily infect tadpoles, disseminate widely in tadpole tissues, and persist up to 50 days. Compared to the mouse infection model, which showed marked and rapid decreases in bacterial abundance from the time of infection within 14 dpi, *X. laevis* tadpoles did not clear the pathogen as rapidly, indicating it could serve as a reliable animal model to study chronic mycobacterial infections. This provides a convenient time period for future investigation.

In addition, the dissemination, expansion, and persistence of *Mab*, especially when inoculated by intraperitoneal injection, did not induce sizable mortality. It is noteworthy that the recovery of bacteria in culture is indicative of an active persisting infection rather than typical latency as with *Mtb* [27]. This is reminiscent of our previous study with *M. marinum*, where we demonstrated that tadpoles show resistance to *M. marinum* by promoting an active tolerance to the pathogen [26]. This system will now permit us to investigate the host immune response to determine whether it is characterized by the induction of anti-inflammatory cytokine (e.g., IL-10) as for *M. marinum* and whether distinct responses are elicited against R and S *Mab*.

It is noteworthy that IP inoculation did affect the survival of the S morphotype in vivo in tadpoles, especially in PLs. Infection of the peritoneal cavity may have induced a more robust innate immune response, resulting in phagocytosis of this morphotype, whereas aggregation of the R morphotype may have allowed it to escape engulfment. Alternatively, R *Mab* may have a better capacity to resist host cell microbicidal activity. In fact, the rapid dissemination of R *Mab* with IP injection in comparison to S *Mab* suggests that R *Mab* is more equipped to persist within phagocytes, which may trigger their rapid dissemination through the organism.

Another notable finding is the unanticipated higher tadpole susceptibility to S *Mab* when inoculated by IC injection. Unlike the zebrafish survival assays, in which mortality is higher in R Mab infected larvae, IC injection of the S morphotype was more lethal than IP injection of S *Mab* [22]. Relatively low doses of S *Mab* (5 × 10^5^ CFUs) were used for the IP and IC injection in tadpoles. Our results suggest that S *Mab* induces an acute infection, resulting in early fatality due to a robust immune response. The R morphotype appears to affect the host during later stages of the infection, supporting its characterization as a persistent pathogen.

In humans, *Mab* can persist in tissues for years without showing symptoms but can reactivate into a systemic infection [16]. After two to three months, *X. laevis* tadpoles undergo metamorphosis, which is accompanied by a drastic remodeling of their immune system [28]. It will be interesting to examine the outcome of persisting S and R *Mab* during this developmental period. Notably, metamorphosis marks the transition between the tadpole adaptive immune system dominated by innate T cells and tolerance to mycobacteria pathogens to an adult adaptive immune system driven by conventional T cells and proinflammatory antimycobacterial host responses [26]. Furthermore, during the metamorphosis transition, T cells are no longer being generated in the thymus and T cell function is suppressed [29]. This suppression of the adaptive immune response, resembling immunocompromised conditions in human patients, may allow the R morphotype to flourish and cause tissue damage. Evaluating the pathogenicity of R and S *Mab* in *X. laevis* tadpoles during metamorphosis, between stages 60 and 66 [30], could provide a unique model to examine *Mab* disease progression in vivo in naturally immunodeficient *X. laevis* hosts. It will also be interesting to determine whether *Mab* infection at the tadpole stage will result in tolerance or pathogenesis after metamorphosis.

## 4. Materials and Methods

### 4.1. Animal Husbandry

Outbred *X. laevis* tadpoles were obtained from our *X. laevis* Research Resources for Immunology at the University of Rochester, New York, USA, (http://www.urmc.rochester.edu/smd/mbi/xenopus/index.htm). All animals during the experiment were carefully handled under the University of Rochester Committee on Animal Resources regulations (approval number 100577/2003-151).

Six-week-old female C57BL/6 mice were purchased from Jackson Laboratories (Bar Harbor, MN). The mice were maintained in the Syracuse Veterans Affairs Medical Center Veterinary Medical Unit in an animal Biosafety Level 2 facility. Mice were housed in micro-isolator cages with a maximum of 8 mice per cage. There was a one-week acclimation period before mice were manipulated in any way. The mice ingested sterile water and Prolab^®^ RMH 3000 rodent chow (PMI Nutrition International, Brentwood, MO, USA.) and libitum throughout the course of the study. The animal protocols, ACORP# 021, were approved by the Veterans Affairs Medical Center’s IACUC (Subcommittee for Animal Studies (SAS), Syracuse, NY, USA).

### 4.2. Mycobacterial Strains and Culture Conditions:

*M. abscessus* S strain PM3044, a smooth colony clone of the type strain ATCC19977, and the R strain PM3492, a *∆mbtH* mutant derived from PM3044 via suicide-vector mediated allelic exchange, were used for all comparisons in mouse and *X. laevis* infections [31]. The R phenotype of PM3492 can be complemented to the wild-type S phenotype by the *mbtH*^+^ gene *in trans* [31]. *Mab* cultures were grown at 37 °C for 5 days in Middlebrook 7H9 broth supplemented with 0.2% glycerol, 10% albumin–dextrose–saline (ADS) supplement, and 0.05% Tween-80. The cultures were then centrifuged, washed with amphibian phosphate buffer (aPBS) with 0.05% of Tween 80, resuspended in 50% aPBS and 50% glycerol, and frozen at −80 °C. After freezing, the number of viable colony-forming units (CFU/mL) was determined. The working concentration of all bacterial samples had to be at or above 10^7^ CFU/mL to be used for tadpole infection. R *Mab* samples were gently sonicated prior to plating and infection to disperse clumps.

To avoid contamination by *X. laevis* microbiota, Middlebrook 7H10 media supplemented with 0.5% glycerol, 10% ADS, 200 μg/mL ampicillin, and 40 μg/mL polymyxin B was used for determination of bacterial burden. Growth of *Mab* on 7H10 media was not affected by the antibiotics.

### 4.3. Mab Mouse Infection Model

*Mab* S strain PM3044 and R strain PM3492 were grown in 7H10 broth with 10% oleic acid–albumin–dextrose–catalase (OADC) supplement and 0.05% Tween 80 on a rotary shaker at 37 °C for 5–7 days. On the day of infection, the organism was diluted to 100 Klett units (corresponding to 5 × 10^8^ CFU/mL) and 0.1 Klett units (5 × 10^5^ CFU/mL). The actual CFU count was determined by diluting the culture to 5 × 10^2^ CFU/mL and plating in triplicate on Cation-adjusted Mueller Hinton agar. The agar plates were incubated in ambient air at 37 ℃ for 5–7 days.

C57BL/6 mice were anesthetized by intramuscular delivery of a telazol (45 mg/kg)/xylazine (7.5 mg/kg) cocktail (Lederle Parenterals, Carolina, Puerto Rico and Bayer Corp., Shawnee Mission, Kansas, respectively) and infected intranasally with approximately 1 × 10^7^ and 1 × 10^4^ viable *Mab* S (PM3044) or R *Mab* (PM3492) in a 20 μL volume.

The actual inoculum of PM3044 was 2.4 × 10^6^ CFU (high) and 2.4 × 10^3^ CFU (low). The inoculum for PM3492 was 2.2 × 10^6^ CFU (high) and 2.2 × 10^3^ CFU (low). At 1, 7, and 14 days postinfection, 4 mice from each group were euthanized by CO_2_ asphyxiation, and their right lung was removed aseptically and homogenized in saline with Tween-80. The homogenates were serially diluted and plated on Mueller Hinton agar to determine CFUs/lung. Four mice were used for each timepoint. Agar plates were incubated in ambient air at 37 °C for 7–14 days.

### 4.4. Mab Inoculation in Tadpoles

Three-week old tadpoles (development stage 52) [30,32], were infected either by intraperitoneal (IP) injection of R or S *Mab* (5 × 10^5^ CFU in 10 μL volume) or by intracardiac (IC) injection (5 × 10^5^ CFU in 5 μL volume). Controls were injected with sterile aPBS. For survival analysis following *Mab* IP inoculation, 20 tadpoles were monitored daily for 40 days. For IC inoculation, 72 tadpoles were monitored for 50 days. Remaining survivors were euthanized, and organs were collected to determine bacterial loads.

### 4.5. Colony-Forming Assay

Peritoneal leukocytes (PLs), liver, spleen, and lungs were collected and lysed by bead beating in 500 μL aPBS. Undiluted and 10× diluted lysates (100 μL out of 500 μL total lysate) were plated on an antibiotic medium. Colony counts were obtained 5 days after incubating the samples at 37 °C. Organ homogenates not used for plating were saved for DNA isolation at −80 °C.

### 4.6. Statistical Analysis

Statistical significance of survival data was determined by a log-rank (Mantel–Cox) test using the GraphPad Prism 8 software (GraphPad Software, Inc., La Jolla, CA, USA). Two-way ANOVA statistical analysis and multiple comparisons analysis followed by using Tukey’s multiple comparisons test was performed to the compare CFU/tissue of R and S *Mab* extracted from organ lysates. A value of *p* > 0.05 was considered significant.

## 5. Conclusions

Our data show that while *Mab* was rapidly cleared in C57BL/6 mice, both S and R *Mab* morphotypes inoculated by intraperitoneal injection in *X. laevis* tadpoles widely disseminated in tissues, including liver and lungs, and persisted in active form for up to 40 days without causing significant mortality. Furthermore, the R morphotype persisted at a higher bacterial load. In contrast, intravenous-like intracardiac inoculation with S *Mab* induced significant mortality compared to that with R *Mab*. These data provide evidence that tadpoles of the amphibian *X. laevis*, which have lungs and functional T cells, can serve as a useful experimental organism complementary to the murine model for persistent *Mab* infection and pathogenesis.

## Data Availability

The data that support the findings of this study are available from the corresponding author upon reasonable request.
